# Ulcerative Colitis as a Novel Cause of Increased Need for Levothyroxine

**DOI:** 10.3389/fendo.2019.00233

**Published:** 2019-04-16

**Authors:** Camilla Virili, Ilaria Stramazzo, Maria Giulia Santaguida, Giovanni Bruno, Nunzia Brusca, Silvia Capriello, Miriam Cellini, Carola Severi, Lucilla Gargano, Marco Centanni

**Affiliations:** ^1^Department of Medico-Surgical Sciences and Biotechnologies, Sapienza University of Rome, Latina, Italy; ^2^Endocrinology Unit, Santa Maria Goretti Hospital, AUSL Latina, Latina, Italy; ^3^Gastroenterology Unit, Department of Internal Medicine and Medical Specialties, Sapienza University, Rome, Italy

**Keywords:** hypothyroidism, levothyroxine absorption, microbiota, ulcerative colitis, IBD

## Abstract

**Background:** Thyroxine absorption takes place at the small intestine level and several disorders affecting this intestinal tract lead to thyroxine malabsorption. An increased need for thyroxine has also been observed in gastric disorders due to variations in drug dissolution and/or in its ionization status. Ulcerative colitis (UC) is an inflammatory bowel disease that has been postulated as a potential cause of the increased need for thyroxine, but there is a lack of evidence on this topic. This study is aimed at measuring the thyroxine requirement in hypothyroid patients with UC.

**Patients and Methods:** Among 8,573 patients with thyroid disorders consecutively seen in our referral center from 2010 to 2017, we identified 34 patients with a definite diagnosis of UC. Thirteen of them were hypothyroid (12 F/1 M; median age = 53 years), bearing UC during the remission phase and in need for thyroxine treatment, thus representing the study group. The dose of T4 required by UC patients has been compared to the one observed in 51 similarly treated age- and weight-matched patients, compliant with treatment and clearly devoid of any gastrointestinal and /or pharmacological interference.

**Results:** To reach the target serum TSH, the dose of thyroxine had to be increased in twelve out of thirteen (92%) hypothyroid patients with ulcerative colitis. The median thyroxine dose required by UC patients was 1.54 μg/kg weight/day, that is 26% higher than the control patients, to reach a similar TSH (1.23 μg/kg weight/day; *p* = 0.0002). Since half of our study group consisted of patients aged over 60 years old, we analyzed the effect of age on the subdivision in two classes. Six out of seven (86%) adult patients (<60 years) required more T4 than those in the respective control group (1.61 vs. 1.27 μg/kg weight/day; +27%; *p* < 0.0001). An increased dose (+17%; *p* = 0.0026) but to a lesser extent, was also observed in all patients over 60 years, as compared to the control group.

**Conclusions:** In almost all hypothyroid patients with UC, the therapeutic dose of thyroxine is increased. Therefore, ulcerative colitis, even during clinical remission, should be included among the gastrointestinal causes of an increased need for oral thyroxine.

## Introduction

The treatment of choice for hypothyroidism, a widespread clinical condition, is represented by oral levothyroxine sodium (LT4) in tablet formulation (1). This drug is characterized by a narrow therapeutic index and thus requires accuracy and correct dose individualization, the mode of assumption and the knowledge of the potential interfering factors. Carelessness about interfering factors may ensue an increased need for T4, a low effectiveness of LT4 and increased health costs as well (2–4). Following gastric dissolution, oral LT4 is incompletely absorbed at the small intestine level (5, 6), probably through several transporters not fully characterized yet (7). Once reaching the target tissues, thyroxine it is metabolized by deiodination (8, 9) and, mainly in the liver, also through the conjugation of the phenolic group and deamination or decarboxylation of the alanine side chain (10). Once deconjugated, T4 may enter the enterohepatic recycling mechanism, although its net effect on the LT4 pharmacological homeostasis has not been fully understood (11, 12). To note, it has been estimated that about 25% of the ingested dose may be found in feces in physiological conditions (13). Several pharmacokinetic and clinical studies enlightened the role of various drugs and gastrointestinal diseases in determining an increased need for LT4, by interfering with the steps key to the efficacy of this treatment (4). Besides the concomitant ingestion of thyroxine and food or drugs (2, 14, 15), the disorders leading to thyroxines increased need have been described in almost every gastrointestinal district. Helicobacter pylori infection (16), chronic atrophic gastritis (17) in the stomach, celiac disease (18), lactose intolerance (19), parasitosis (20), pancreatic insufficiency (21), and short bowel syndrome (22) in the small intestine, were all associated with oral T4 refractoriness. In addition, esophageal dysmotility (23) and liver cirrhosis (24) have been involved in affecting the efficacy of oral thyroxine. Concerning the bowel, evidence is scarce: a review from Liwanpo and Hershman (14) reported inflammatory bowel diseases (IBD) as a possible cause of increased need for thyroxine but without definite evidence. Now, the acronym IBD encompasses Crohn's disease and ulcerative colitis (UC), multifactorial immune-related diseases with different clinical presentation; Crohn's disease may affect all the gastrointestinal tracts whereas ulcerative colitis primarily affects the colon and the rectum and the cases of backwash ileitis are rare (25). These diseases may concur with Hashimoto's thyroiditis (HT), the most common cause of hypothyroidism, in polyglandular autoimmune syndrome type III b (26), since Hashimoto's thyroiditis and Crohn disease are CD4+T helper (Th) 1-polarized (27–30) disorders, while UC is an atypical Th2-polarized disease (27). In a paper by Benvenga et al. (3), only one case of Crohn's enteritis as a cause of the increased need for levothyroxine has been mentioned. On the contrary, whether UC may interfere with LT4 treatment efficacy is not known. The aim of this study is to analyze the possible role of UC in determining an increased need for oral T4 in a large cohort of consecutively-examined patients with thyroid disorders.

## Patients and Methods

### Design of the Study

According to the aim of the present study, we have retrospectively analyzed the clinical records of 8,573 patients with thyroid disorders seen in our referral center in Sapienza University of Rome, Endocrinology Unit, Latina, Italy, from 2010 to 2017. Forty-two patients with IBD were detected. Thirty four patients had a definite diagnosis of UC and among them 13 patients met the following inclusion criteria: (a) hypothyroidism due to Hashimoto's thyroiditis in need of treatment with LT4 according to ATA Guidelines (1); (b) thyroxine treatment in tablet formulation with the same dose and brand over at least 2 years; (c) stable TSH values between 0.8 and 2.5 mU/l in at least two subsequent control visits; (d) UC in a remission phase since at least two years, with a stable low dose treatment of mesalazine (≤ 2 gr/day). The phase of UC was evaluated by the Partial Mayo Score, a simplified index evaluating patient's perception of disease activity that shows a good correlation with endoscopy findings (31).

**Figure 1:**
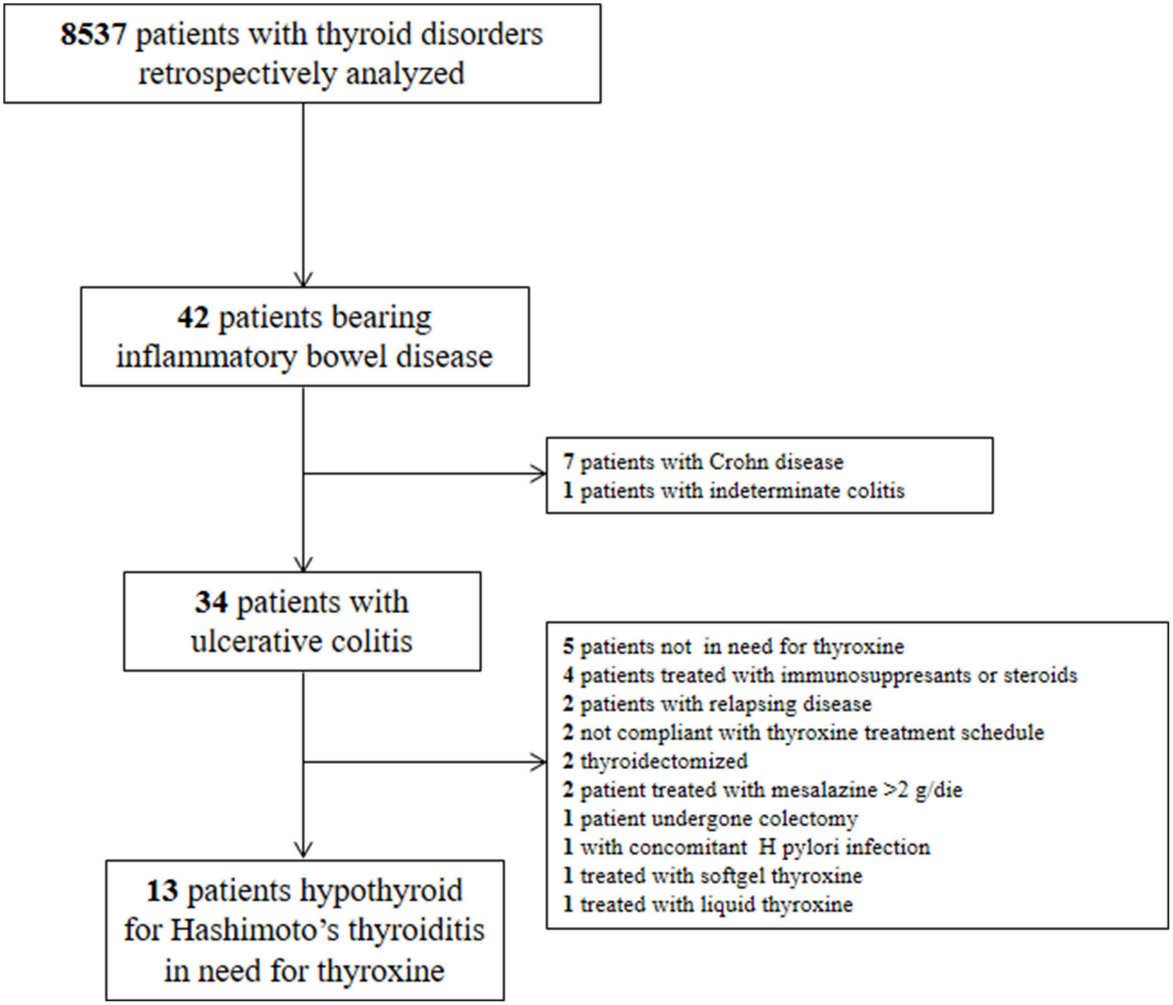
Diagram of patients' selection according to inclusion and exclusion criteria.

We excluded: (a) pregnant or lactating women (b) patients with active or relapsing UC; (c) patients treated with immunosuppressants, steroids and/or with drugs interfering with thyroid pharmacologic homeostasis (12); (d) patients not compliant with the thyroxine treatment schedule or using softgel or liquid thyroxine formulation, for the possible pharmacokinetic bias (32–35) (e) bearing further chronic, infectious, inflammatory, or neoplastic diseases. In particular, we excluded patients with the presence and/or signs or symptoms suggestive of H. pylori-related gastritis, atrophic gastritis, celiac disease, and/or previous gastrointestinal surgery, all conditions leading to an increased need for oral thyroxine (36). We also excluded thyroidectomized patients due to the higher doses they often need (37). The diagram of patients' selection is depicted in Figure 1.

According to the policy of our Center, all patients pledged to take thyroxine in fasting conditions, abstaining from eating or drinking for at least 1 h. All patients enrolled in the study met these requirements. Furthermore, in all patients we checked the compliance with LT4 treatment by specifically interviewing patients at every control visit. Patients with suspected poor adherence to therapy (e.g., fluctuating TSH values in two consecutive analysis, inconsistent answers to interview, etc.) were excluded.

To calculate the possible excess of thyroxine required by our patients, we compared their individual T4 requirement with the one observed in 51 similarly treated age- and BMI-matched patients, clearly devoid of gastrointestinal and/or pharmacological interference. We considered a significantly increased requirement for thyroxine stably higher (at least two times) than 15% of the dose needed by the reference group.

### Diagnosis of Hashimoto's Thyroiditis

The diagnosis of HT was based on the presence of at least two of these three criteria: ultrasonographic features highly suggestive of HT, the presence of hypothyroidism, and high titers of anti-thyroperoxidase antibodies (TPOAb).

### Diagnosis of Ulcerative Colitis

The diagnosis of UC was based on clinical grounds and supported by findings from endoscopic and histological analysis (38).

### Laboratory Measurement

Serum TSH, FT4 and serum anti-thyroid peroxidase antibodies (TPO-Ab) levels were assayed by RIA commercial kits (Brahms, Henningdorf BEI, Berlin, Germany).

### Statistical Analysis

Daily thyroxine requirements in the control and study group were compared by Mann-Whitney test for non-parametric data and expressed as median values (IQ1-IQ3). Statistical analysis was performed by the GraphPad version 5.0 for Windows (GraphPad Prism, La Jolla CA, USA).

## Results

**Figure 2:**
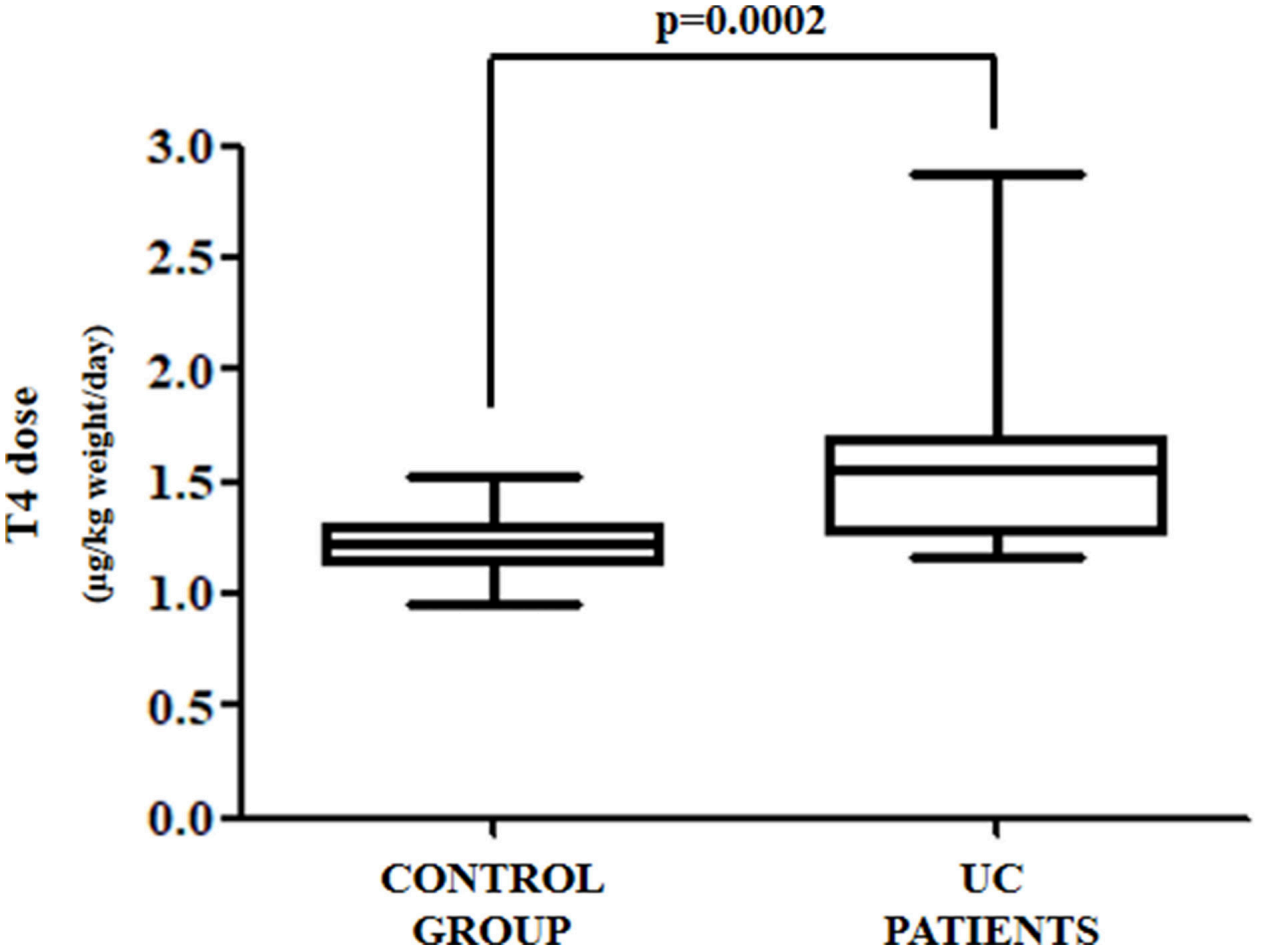
Daily thyroxine requirement in all patients affected by UC as compared to control group patients.

**Figure 3:**
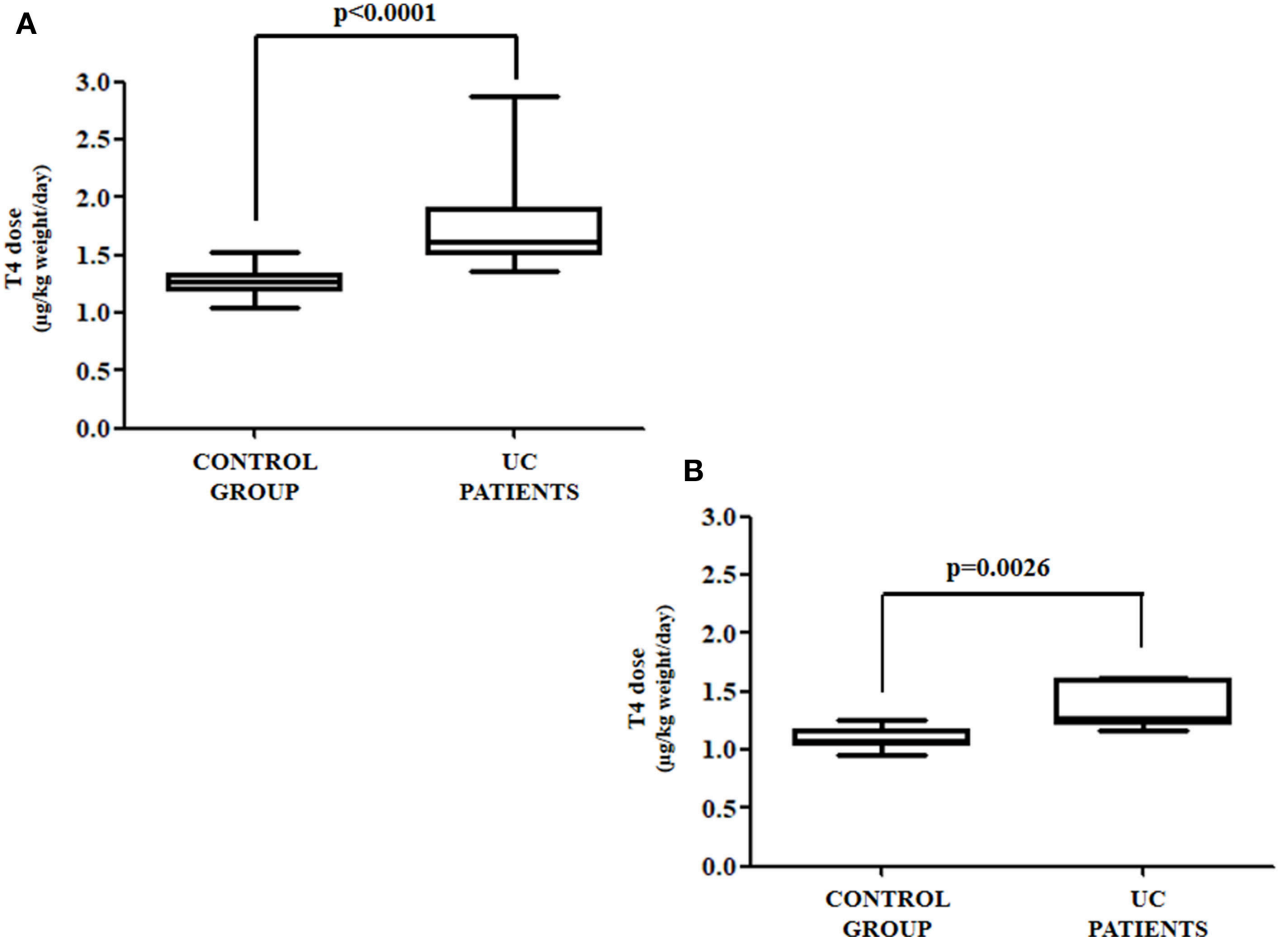
**(A)** Thyroxine requirement in UC and control group patients under the age of 60; **(B)** thyroxine requirement in UC and control group patients aged over of 60 years.

The anthropometric and biochemical characteristics of the control and the study group are summarized in Table 1. All patients with UC reached target TSH (0.8–2.5 mU/L) requiring a median thyroxine dose of 1.54 μg/kg weight/day. Such a daily requirement was 26% higher than in the control group, in order to reach a similar TSH (1.23 μg/kg weight/day) (Figure 2). Since half of our study group consisted of patients aged over 60 years, and since elderly patients usually have a reduced daily thyroxine requirement, we analyzed the effect of age on the subdivision in the two classes. Therefore, the patients in both the control and study groups were subdivided into adults (aged less than 60 years) and seniors (aged more than 60 years). In the control group, 35 were adult patients [median age = 40 (32–45) years] and 16 senior patients [median age = 63 (61–70)]. Seven out of the 13 patients with UC were adult [median age = 47 (35–50) years] and six senior patients [median age = 66 (63–71)]. Six out of seven (86%) adult patients required more T4 than the those in the respective control group. Therefore, a significant increase of 27% of the median T4 dose in adult patients with UC (1.27 vs. 1.61 μg/kg weight/day) was observed, as compared with those in the control group (Figure 3A). All senior patients showed an increased need for thyroxine but to a lesser extent. The median dose in this group was, in fact, significantly higher than its own control (1.07 vs. 1.25 μg/kg weight/day) (+17%) (Figure 3B). Overall, the analysis of these data revealed that in twelve out of thirteen (92%) patients with HT also bearing UC, the therapeutic dose of thyroxine had to be increased.

**Table 1:** Anthropometric and biochemical characteristics of control and study group.

	**Control group**	**UC Patients**	***p***
*n*	51	13	
Age (years)	45 (36–61)	53 (47–65)	0.1583
Sex (M/F)	3/48	1/12	
Weight (Kg)	61 (57–70)	63 (60–80)	0.4085
Height (cm)	162 (1.59–1.67)	164 (1.62–1.66)	0.4686
BMI (Kg/m^2^)	22.67 (21.14–24.68)	23.56 (22.70–24.43)	0.6298
TPO Ab (UI/l)	258 (158–690)	313 (127–631)	0.7540
Last assayed TSH (mU/l)	1.10 (0.79–1.49)	1.13 (0.58–1.30)	0.5647
Daily dose (μg/day)	75 (74–86)	100 (100–107)	<0.0001

## Discussion

Our data clearly indicates that in almost all hypothyroid patients affected by UC, an increased need for thyroxine is observed, even during the remission phase of the UC. This increase was recognized both in adult and older patients, being the extent lower in the latter group. To the best of our knowledge, this is the first report of an increased need for thyroxine in UC patients. The malabsorption of other drugs in patients with UC has also been examined in a few studies but the results obtained were contrasting: in particular, a study carried out in patients with acute UC reported a delayed absorption of prednisolone, while there was no evidence of an impaired absorption of metronidazole, as compared to healthy volunteers (39).

Understanding the mechanism of this effect of the UC on the T4 dose in hypothyroid patients is not an easy task. In fact, UC during its acute phase mainly, involves full or sectional bowel tracts (40), where absorption of thyroxine does not take place (5). In addition, our patients were all in the remission phase of UC, when the mucosal damage and leakage of fluids and solutes should supposedly be on its end. One possible explanation comes from the findings of several reports indicating that additional pathologic features accompany the main bowel damage (41–43). Indeed, an increased permeability of small intestine even during the remission phase (41) as well as small intestinal reduced transit time, alterations in electrolytes, and solute and nutrient trafficking has been described in UC patients (42, 43). Furthermore, these alterations may lead to small intestinal bacterial overgrowth that are bound to nutrient and vitamin malabsorption (44) and to hypothyroidism (45).

Noticeably, all patients of our study group were treated with low doses of mesalazine to maintain the disease remission; this fact may raise the doubt that the increased need for thyroxine might be due to interference by other mechanisms. In fact, mesalazine is an anti-inflammatory non-steroidal drug that belongs to the class of 5-aminosalicylic acid compounds, used in the treatment of active mild to moderate forms of UC and in the maintenance of remission. Since this compound is a salicylate, it has the ability to displace the iodothyronines from the plasmatic binding proteins (46), thus possibly interfering with thyroxine homeostasis in the blood. Indeed, an interesting report demonstrated a striking reduction of FT4 levels in patients taking salsalate, a dimer of salicylic-acid (47). Surprisingly enough, however, after 3 weeks of treatment the thyroxine homeostasis was restored (47). Our patients were all treated with low-doses of mesalazine in a formulation in which the active ingredient is coated with a methacrylate copolymer (Eudragit-S), which dissolves at pH ≥ 7. This fact limits the systemic absorption at the small intestine level while optimizes its delivery at the colic mucosa level (48). Indeed, mesalazine may exert its prevailing therapeutic effect by local topical activity (i.e. reducing the formation of prostaglandins and leukotrienes and inhibiting T cell activation and proliferation) (48). The scarce systemic absorption of this drug weakens the hypothesis of a possible effect on thyroid pharmacological homeostasis.

This study has some limitations: first of all, the sample size is low. This is because UC is a rare disease, is relapsing and subject to not avoidable treatments, known to interfere with the absorption and/or the metabolism of thyroid hormones. Second, as a retrospective study, it may contain some biases due to the duration of hypothyroidism, although anthropometric, clinical and biochemical characteristics were superimposable both in the control and in the study group (Table 1).

## Conclusions

The present findings allows for the inclusion of ulcerative colitis, even during clinical remission, among the causes of an increased need for oral thyroxine. Therefore, despite the fact that the UC's lesions do not seem to directly affect thyroxine absorption sites, the presence of this disease in hypothyroid patients warrants a careful individualization of treatment with oral thyroxine.

## Ethics Statement

The study was carried with written informed consent from all participants and data collected remained strictly confidential and anonymous, according to the ethical rules of Sapienza University of Rome, and adhering to the guidelines in the Declaration of Helsinki.

## Author Contributions

CV and MaC conceived and designed the study. IS, MS, and SC analyzed the data. MiC, NB, LG, GB, and CS critically reviewed the results. CV and MaC wrote the paper. All authors contributed to revise the manuscript.

### Conflict of Interest Statement

MC has been a consultant for Akrimax Pharmaceuticals, Cranford, NJ, USA and received honoraria and travel expenses to attend advisory boards meetings, and from Institut Biochimique SA (IBSA), Lugano, CH to attend International Meetings. The remaining authors declare that the research was conducted in the absence of any commercial or financial relationships that could be construed as a potential conflict of interest.
